# Evaluation of the Demographics, Clinical Laboratory Parameters, and Outcomes of Hospitalized Oncological Versus Non-oncological COVID-19 Patients

**DOI:** 10.7759/cureus.73313

**Published:** 2024-11-09

**Authors:** Ilona Georgescu, Stefan Alexandru Artene, Lucian-Ion Giubelan, Daniela Elise Tache, Florentina Dumitrescu, Carmen Duta, Adina Andreea Mirea, Elena-Victoria Manea (Carneluti), Anica Dricu, Oana Stefana Popescu

**Affiliations:** 1 Biochemistry, Faculty of Medicine, University of Medicine and Pharmacy of Craiova, Craiova, ROU; 2 Infectious Diseases, "Victor Babeş" Clinical Hospital of Infectious Diseases and Pneumo-phtisiology, Craiova, ROU; 3 Biochemistry, Faculty of Medicine, “Carol Davila” University of Medicine and Pharmacy, Bucharest, ROU; 4 Dental Medicine, University of Medicine and Pharmacy of Craiova, Craiova, ROU

**Keywords:** covid-19, demographic, non-oncological, oncological, pandemic

## Abstract

Introduction

The COVID-19 pandemic emerged globally in 2019, exposing healthcare vulnerabilities. This study delves into the impact of COVID-19 on cancer patients, a high-risk group with increased susceptibility and mortality rates. Recent research underscores cancer patients' vulnerability to severe disease, often due to compromised immunity.

Materials and methods

This retrospective study analyzed data from 474 adult COVID-19 patients, admitted between March 2020 and July 2023. Patients were categorized into two groups: those with a medically recorded oncological disease (237) and those without any malignant history (237). Demographic and hematologic analysis aim to unveil COVID-19 impact on individuals with cancer history.

Results

Statistically significant differences in blood parameters highlighted distinctions, with cancer patients exhibiting higher creatinine, leukocyte, and D Dimers levels as well as lower hemoglobin, neutrophile, lymphocyte, and Serum Glutamate-Pyruvate Transaminase (SGPT) levels. Non-significant differences in certain parameters prompted a nuanced exploration of metabolic and coagulation variations.

Conclusion

This study unveils global COVID-19 effects on cancer patients, emphasizing clinical and laboratory differences. Findings underscore the imperative need for targeted interventions and enhanced support for cancer patients during the pandemic. Study limitations stress careful interpretation, urging further exploration of COVID-19 and cancer interplay.

## Introduction

COVID-19, caused by SARS-CoV-2, has rapidly become a major global challenge since its emergence in 2019 [1,2]. This respiratory infection exposed vulnerabilities in health care systems and led to a major overhaul of medical practice around the world. Infections range in severity from asymptomatic or mild to severe symptoms, often requiring intensive medical intervention. Of particular concern are high-risk groups such as the elderly and people with co-morbidities, including cancer patients [3,4]. These patients are prone to severe complications and often have a high mortality rate, highlighting the importance of an individualized approach to infection control in this category of patients [5].

Regarding the relationship between COVID-19 and cancer, recent studies have shown that cancer patients are at a significantly higher risk of developing severe disease and therefore have higher mortality rates than the general population [6]. This increased susceptibility is often associated with a weakened immune system, either as a result of the cancer itself or previous treatments such as chemotherapy or radiation therapy. Additionally, cancer-specific treatments may impact the body's ability to respond effectively to SARS-CoV-2 infection, further complicating the treatment of both health conditions simultaneously [7,8]. In this context, careful monitoring and continuous adjustment of treatment strategies are essential to provide optimal care to cancer patients affected by COVID-19.

The interaction between COVID-19 and cancer treatment is an important area in the treatment of patients affected by both diseases. For example, administration of corticosteroids, a key component in the treatment of severe forms of coronavirus disease (COVID-19), can help control excessive inflammatory responses. However, the side effects and risks of these drugs must be considered, especially their impact on cancer progression [9,10]. Corticosteroids can impair the immune system and change its response to cancer cells, so a careful approach is needed when administering these drugs to cancer patients [11]. At the same time, anti-tumor therapies that primarily target the immune system to fight cancer cells may also influence the body's response to SARS-CoV-2 infection [12,13]. This creates a delicate balance between treating cancer and treating viral infections. Given the large differences in cancer types and the specific conditions of each patient, it is essential to develop individualized treatment protocols that take this complex interaction into account. Therefore, the importance of an integrated and individualized approach to maximize treatment efficacy and minimize the risks associated with co-treatment of COVID-19 and cancer is highlighted. Ongoing research and collaboration between infectious disease and oncology experts are required in order to develop precise protocols that address patient-specific needs and provide patients faced with these complex health challenges with the best chance of recovery and survival [14,15].

The overlapping mechanisms between SARS-CoV-2 infection and receptor tyrosine kinases (RTKs) dysregulation in cancer have also been reported by several research groups [16]. In regard to cancer, it is well known that cancerous cells are sensitive to receptor tyrosine kinases (RTKs) inhibition. Given their crucial role in cancer cell survival, different inhibitors for the different types of RTKs have been developed for targeted cancer therapies [17]. Surprisingly, several research studies have pointed out that inhibition of several RTKs (e.g. epidermal growth factor receptor (EGFR), insulin-like growth factor (IGF)-1R) decreases the risk of death in COVID-19 patients [18,19].

The aim of this retrospective study is to analyze the difference between patients with a prior oncological diagnosis and patients with no record of cancer, who are admitted with a COVID infection, in terms of the main hematologic parameters recorded during admission. Through this study, we hope to better understand how oncological patients were affected by the global pandemic and if there were specific areas where the burden was greater in comparison to non-oncological patients to better understand and adjust specific medical approaches for the aforementioned patients.

## Materials and methods

Study design

**Figure 1:**
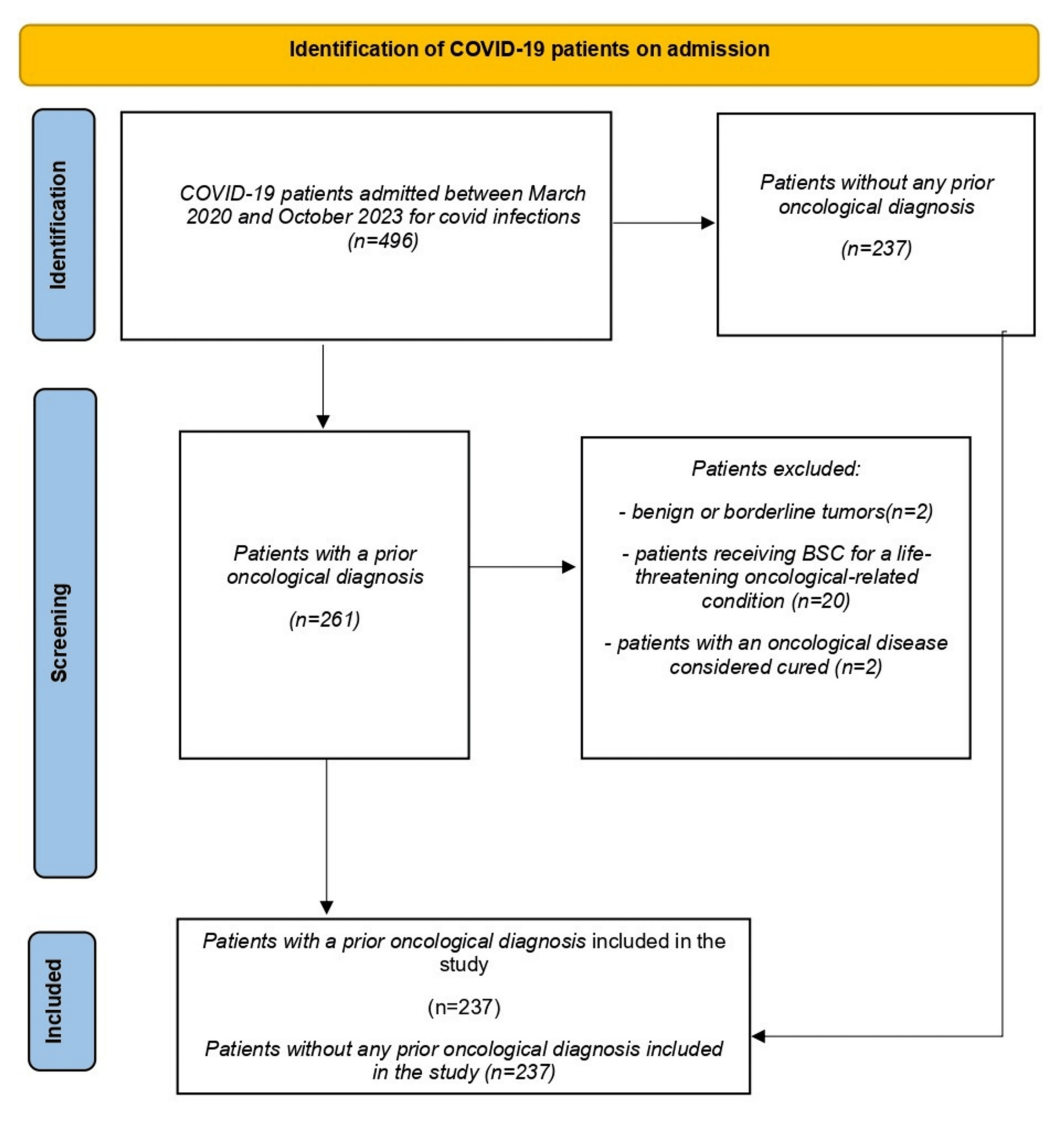
Flowchart for patient selection

Four hundred and seventy-four (n=474) adult patients who were admitted between March 2020 and July 2023 for COVID infection in the coronavirus care unit of the “Victor Babeş” Infectious Disease Hospital of Craiova in Craiova, Romania based on the World Health Organization (WHO) criteria [20]. Of these, 237 had an active oncological disease at the time of admission and were matched on a 1:1 ratio with patients who did not present any record of an oncological disease (237). Patients with benign or borderline tumors were excluded from the study. Similarly, patients with an oncological disease considered cured by standard guidelines, without any major cancer-related comorbidities, were excluded due to their long-term prognosis being similar to patients without a history of cancer. No exclusion criteria were used regarding the histological type, localization, stage or treatment type for the patients who suffered from malignant tumors. Patients from both groups who received the best standard of care (BSC) for a life-threatening cancer-related condition or any other terminal non-oncological disease before being diagnosed with a positive coronavirus test were excluded from the study. No other exclusion criteria were applied for patients in the control group. As such, 261 patients were initially identified and went to the screening process. Of these patients, we excluded 20 patients receiving BSC, two patients with a benign or borderline tumor (one case of ovarian borderline cystadenoma and one case of focal nodular hyperplasia), and two cases of patients with cancers considered cured (one case who received chemotherapy and surgery for breast cancer 14 years ago and a patient who received surgery for early stage melanoma 21 years ago). Patient allocation is represented in Figure 1.

Admission criteria

Cases were divided on admission based on the following criteria:

Mild forms: cases with non-life-threatening signs and symptoms, no oxygen requirements and no radiological signs of pneumonia;

Moderate forms: cases with non-life-threatening signs and symptoms, no oxygen requirements and with radiological signs of pneumonia;

Severe forms: cases with life-threatening signs and symptoms such as respiratory distress, hypoxia (peripheral oxygen saturation (SpO2)<93%), abnormal respiratory gas levels (partial pressure of oxygen (PaO2)< 60 mmHg, partial pressure of carbon dioxide (PaCO2)>50 mmHg);

We elected to exclude critical COVID-19 patients admitted directly into the ICU before enrolment, such as patients that suffered from shock and respiratory failure which required mechanical ventilation or organ failure which required constant ICU monitoring and therapeutic intervention.

Data collection and statistical analysis

Age, sex, origin (urban/rural) and hospitalization length were recorded from the medical charts. The data was reviewed by two physicians licensed in infectious diseases and oncology, respectively, before being recorded in our database.

The following laboratory results performed during hospitalization were taken into consideration: Hemoglobin (Hb), Leucocytes, Neutrophils, Monocytes, Lymphocytes, Thrombocytes, C-Reactive Protein (CRP), Procalcitonin (PCT), Urea, Creatinine, Blood Glucose, Serum Glutamate-Pyruvate Transaminase (SGPT), Prothrombin Time (PT), Prothrombin Index (PI), International Normalized Ratio (INR), D-Dimers (DD), sodium (Na), potassium (K) and pH. The laboratory work was in accordance with standard hospital protocols.

IBM SPSS Statistics version 26.0 was used to conduct statistical analysis (IBM Corp., Armonk, USA). Continuous variables were shown as mean and standard deviation in a descriptive analysis. Both absolute numbers and percentages were used to represent categorical variables. Using the appropriate parametric (t-test) or nonparametric (Mann-Whitney test) tests for quantitative variables and the Chi-squared test for categorical variables, the laboratory results for the oncological and non-oncological controls were compared. To calculate the impact of oncological disease on the previously indicated laboratory markers, logistic regression was employed. P-values of less than 0.05 were deemed statistically significant.

All patients signed an informed consent form before being included in the study, and it was carried out in accordance with the Helsinki Declaration and authorized by the ethics committee of the University of Medicine and Pharmacy of Craiova (nr. 51/10.02.2023).

The study aims to provide high confidence in its results by using a sample size that ensures valid and applicable conclusions in a clinical context. A power level of 0.80 (80%) is desired, indicating an 80% probability of detecting a real effect if it exists, thus minimizing the risk of a Type II error.

The power analysis determined that a sample of 237 oncological patients and 237 non-oncological patients with COVID-19 is sufficient to detect a moderate effect size with a significance level of 0.05 and a power of 0.80. This sample size also offers an additional safety margin, enhancing the reliability of the study's findings and the ability to detect true differences between the two groups. This approach aligns with best practices in clinical and epidemiological research, facilitating valid and applicable conclusions.

The t-test for independent samples was used to compare mean scores between the groups. Before applying the t-test, normality was checked using the Shapiro-Wilk test and homogeneity of variances using Levene's test. The t-test compared the mean of continuous variables, such as inflammatory marker levels, length of hospitalization or recovery rate, between oncological and non-oncological patients with COVID-19.

Additionally, logistic regression analysis was performed to investigate predictive factors for severe COVID-19 outcomes based on oncological status. This analysis adjusted for potential confounding variables, including age, sex and comorbidities.

The use of GraphPad Prism (GraphPad, San Diego, USA) for conducting tests and creating graphs allowed for rigorous statistical analysis and clear visualization of the results. The choice of statistical tests was based on the nature of the data and the research questions, and the verification of assumptions ensured the validity of the applied tests. Adjustments for multiple comparisons were made to maintain the integrity of the results. This transparent and detailed methodology ensures the reproducibility and reliability of the study's conclusions. The appropriate sample size and statistical power provide high confidence in the study's conclusions, allowing for robust and applicable results in practice.

## Results

**Figure 2:**
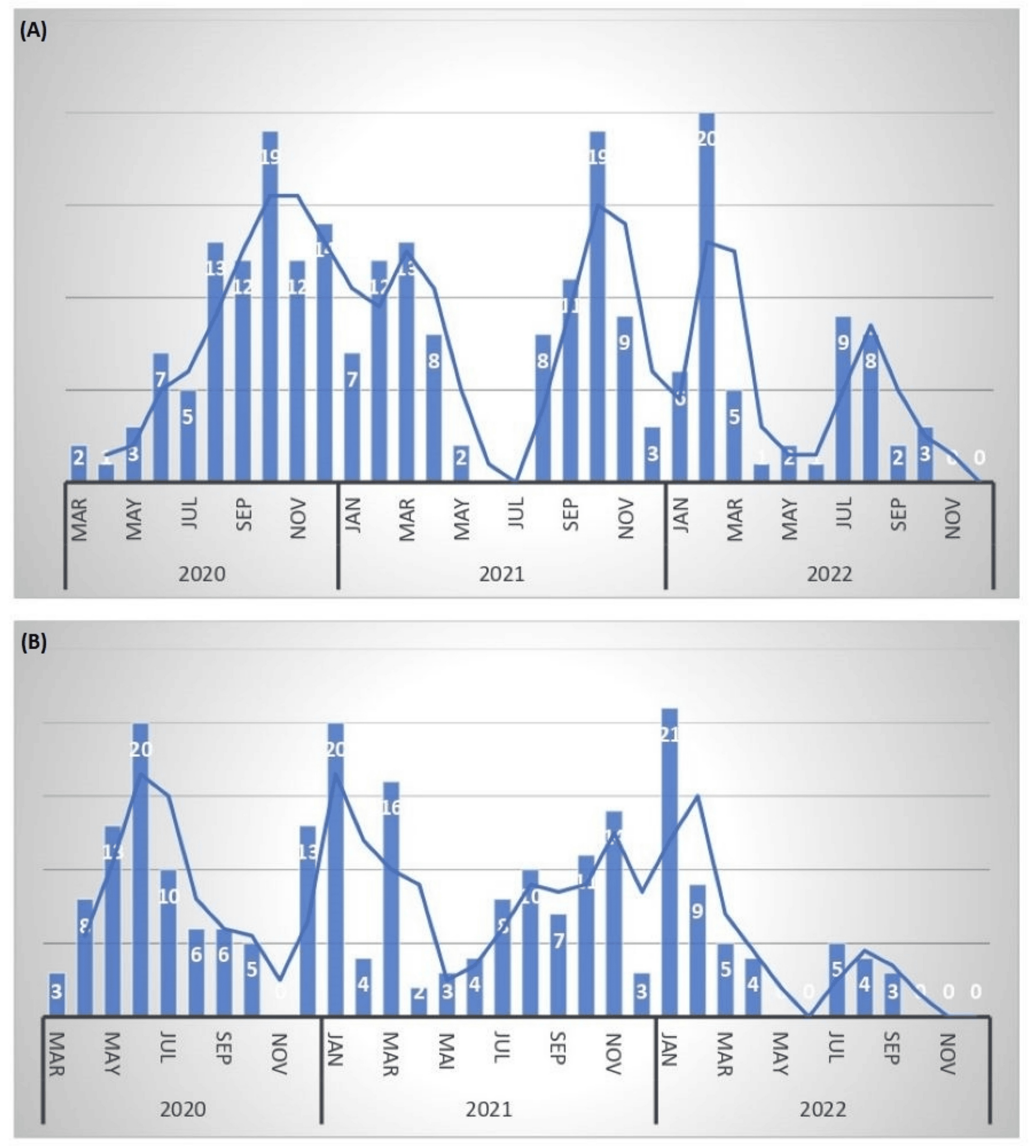
Rate of recruitment of oncological (A) versus non-oncological (B) patients between March 2020 and November 2022.

Demographics of the patients with or without cancer diagnosed with COVID-19 were collected. A total of 474 patients with COVID-19 were included in our analysis in accordance with the selection criteria. Of these, 237 had a previously diagnosed malignant disease while 237 of these had no prior cancer in their records and, as such, were used as a control group. The rate of recruitment for both groups is presented in Figure 2.

**Figure 3:**
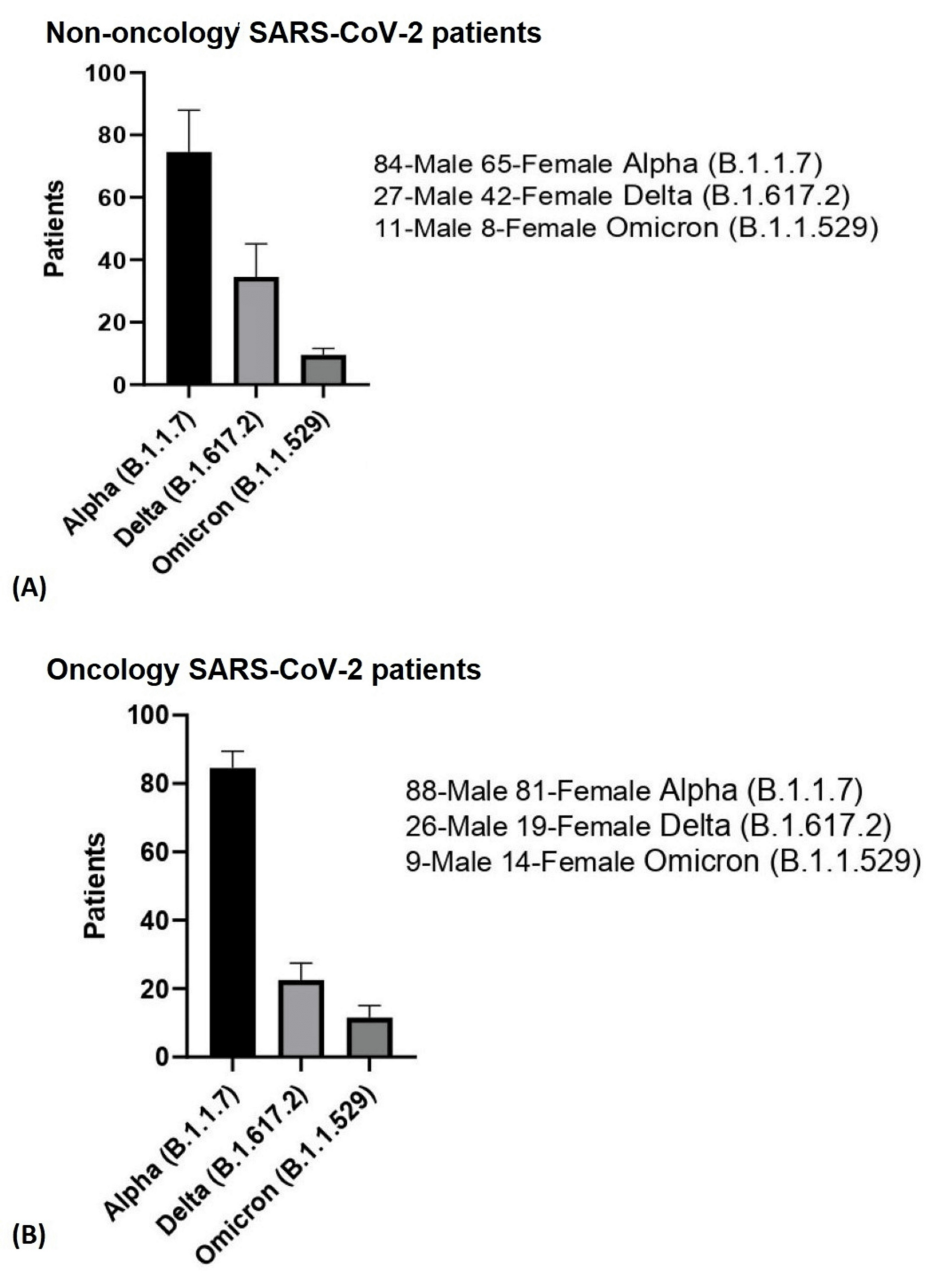
Patient distribution based on the main viral strain at the time of admittance. A. Non-oncological SARS-CoV-2 patients; B. Oncological SARS-CoV-2 patients

The patients were separated based on the most dominant viral strain at the time of admittance (Figure 3).

We then analyzed the main epidemiological characteristics of the patients in both groups. For the oncological group, the mean age was 65.51 years (±12.13 standard deviation (SD)), 51.48% were male, 48.52% were female and 67.51% hailed from an urban area, versus 32.49% who belonged to a rural settlement. The patients in the control group had a mean age of 61.02 years (±14.40 SD), 53.58% were male, 46.42% were female and 74.26% belonged to an urban area versus 25.74% who hailed from a rural community. In terms of COVID-19 infection, we compared the two groups based on admission status, hospitalization length and outcome. Regarding the patients with a recorded malignant disease, 26 (11%) patients were asymptomatic at admission, 43 (18.1%) patients presented a mild form, 92 (38.8%) patients presented a moderate form and 76 (32.1%) patients presented a severe form of disease. For the control group, four (1.69%) were asymptomatic, 45 (18.99%) presented a mild form, 112 (47.26%) presented a moderate form and 76 (32.07%) patients presented a severe form. Patients with a recorded malignant disease had a median hospitalization stay of 11.94 days (±5.28 SD) while patients from the control group presented a median hospitalization length of 11.76 days (±6.28 SD). In terms of outcome, 86 (36.29%) of patients with a malignant disease were considered cured on discharge, 124 (52.32%) were discharged with an improved status, five (2.11%) were discharged with a worsened condition while 22 (9.28%) died during hospitalization. Regarding the patients in the control group, 104 (43.88%) were considered cured on discharge, 119 (50.21%) were discharged with an improved status, four (1.69%) were discharged with a worsened condition and 10 (4.22%) died during hospitalization.

**Figure 4:**
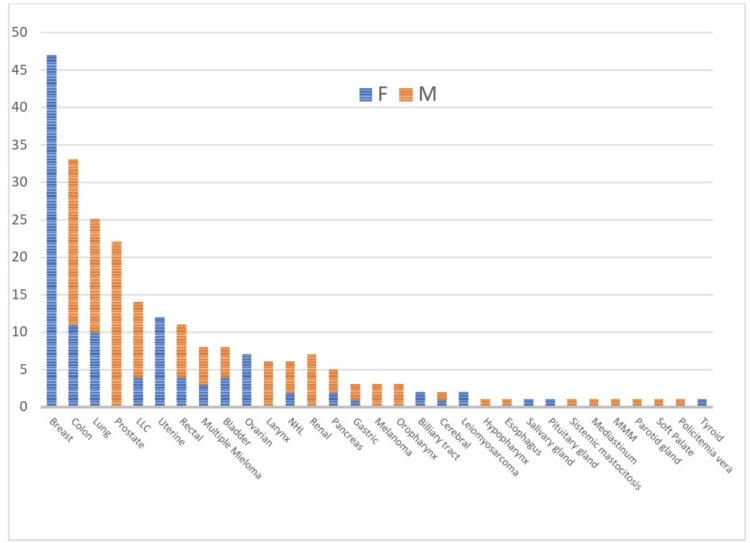
Cancer types of patients included in the study. CLL-chronic lymphocytic leukemia; NHL-Non-Hodgkin Lymphoma; MMM-Myeloid metaplasia with myelofibrosis.

In terms of histopathological origin of cancer, the main types are presented in Figure 1. We observed that the incidence was in accordance with the latest Global Cancer Observatory (GLOBOCAN) statistics regarding cancer incidence and prevalence worldwide and in Europe [21]. As such, the most prevalent were breast cancers (n=47), followed by colon cancers (n=33), lung cancer (n=25), prostate cancer (n=22) and Chronic Lymphoid Leukemia (CLL) (n=14) (Figure 4). Of these, 86.92% presented either a locally advanced, non-operable cancer or metastatic disease.

We further recorded and analyzed the baseline characteristics (age, sex and severity of COVID-19) and the main blood laboratory parameters, and compared them between the oncological and non-oncological groups of patients (Table 1).

**Table 1:** Baseline characteristics (age, sex and severity of COVID-19) and blood parameters for the oncological vs. non-oncological patients M/F-male/female; CRP-C Reactive Protein; PCT-Procalcitonin; SGPT-Serum Glutamate-Pyruvate Transaminase, PT-Prothrombin Time, PI-Prothrombin Index, INR-International Normalized Ratio, DD-D-Dimer, Na-sodium, K-potassium.

Baseline characteristics and blood parameters	Non-Oncological (n=237)	Oncological (n=237)	P-value (two-tailed)		
Age	60.76	65.51		p<0.0001	
Sex (M/F)	0.5169	0.5127		p=0.93	
Severity of COVID-19 (Mild/Severe/Asymptomatic)	1.030	1.113		p=0.23	
Hemoglobin	13.8	12.13		p<0.00001	
Total leukocyte	7204.39	10937.21		p=0.03	
Neutrophil	69.36	66.02		p=0.01	
Monocyte	6.54	6.22		p=0.2	
Lymphocyte	26.44	23. 33		p=0.015	
Thrombocyte	235661.18	222637.13		p=0.18	
CRP	4.61	4.78		p=0.79	
PCT	0.38	1.6		p=0.09	
Urea	43.97	45.96		p=0.52	
Creatinine	2.77	1.18		p<0.00001	
Blood glucose	138.99	127.18		p=0.02	
SGPT	150.41	40.87		p<0.0008	
DD	837.4	1314		p=0.0062	
INR	1.31	1.39		p=0.85	
PT	14.61	15.54		p=0.56	
PI	77.85	73.7		p=0.44	
NA	135.6	131.9		p=0.80	
K	4.1	3.99		p=0.42	
Ph	7.41	7.41		p=0.57	

Non-oncological patients had a mean age of 60.76 years, while oncological patients had a higher mean age of 65.51 years. The analysis revealed a statistically significant difference in the mean age between the two groups (p<0.0001). Regarding the sex distribution between the groups, there was no significant difference (p=0.93). The severity of COVID-19, categorized as mild, severe, or asymptomatic, showed no significant difference between the two groups (p=0.23). Non-oncological patients had a mean severity score of 1.030, whereas oncological patients had a slightly higher mean score of 1.113.

The mean hemoglobin (Hb) levels for the oncological patients were 12.13 g/dl versus non-oncological patients who presented superior mean values of 13.8 g/dl. As represented, we observed a strong statistically significant difference in terms of total Hb levels between the two groups (p<0.00001). We then analyzed and compared leucocyte levels between the two groups. Mean values for non-oncological patients were 7,2x109/L versus 11x109/L for oncological patients with the difference being statistically significant (p<0.05). When we further analyzed the difference between the three main leucocyte factions (neutrophils, monocytes and lymphocytes), we observed two different outcomes. Regarding neutrophil levels, a statistically significant difference between the oncological and non-oncological patients was observed (69% versus 66%)(p<0.05). With regards to monocytes, no difference was observed between the two groups (p=0.2). Lymphocyte levels, however, differed between the two groups, with oncological patients having lower mean levels in comparison to non-oncological patients (26% versus 23%) (p<0.05). In terms of thrombocyte levels, the differences between the mean values of the two groups were practically non-existent (232x109/L versus 235x109/L), thus being considered non-statistically significant (p>0.05). We also calculated the Monocyte/Lymphocyte (M/L), the Thrombocyte/Lymphocyte (T/L) and the Neutrophil/Lymphocyte (N/L) ratios.

**Figure 5:**
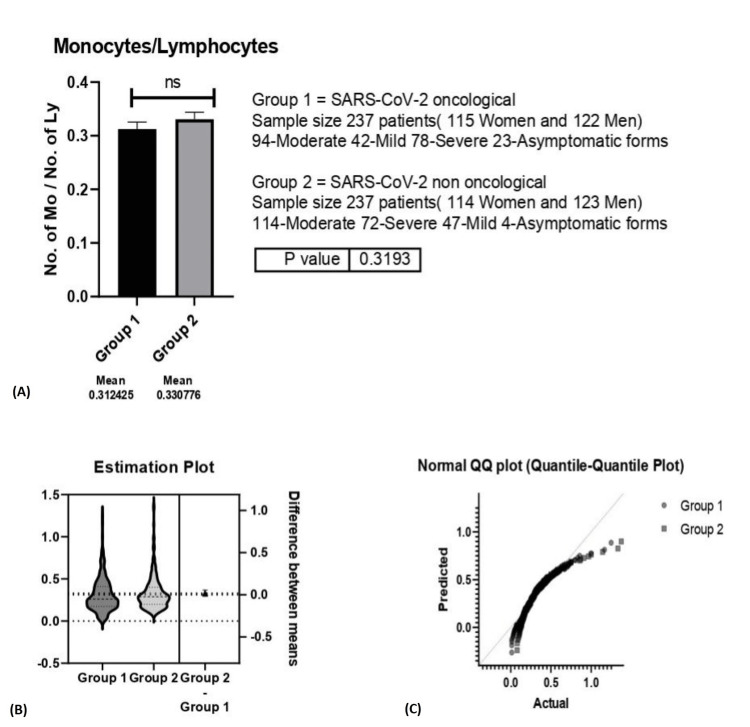
A. Monocyte/Lymphocyte (M/L) ratio between group 1 (oncological patients with COVID-19) versus group 2 (non-oncological patients with COVID-19); B. Estimation Plot for M/L ratio; C. Normal Quartile-Quartile plot for the M/L ratio. A. ns-p>0.05

Taking into consideration the sample size, gender distribution and severity of disease the difference between the oncological and non-oncological patients was not statistically significant (p=0.31) (Figure 5).

**Figure 6:**
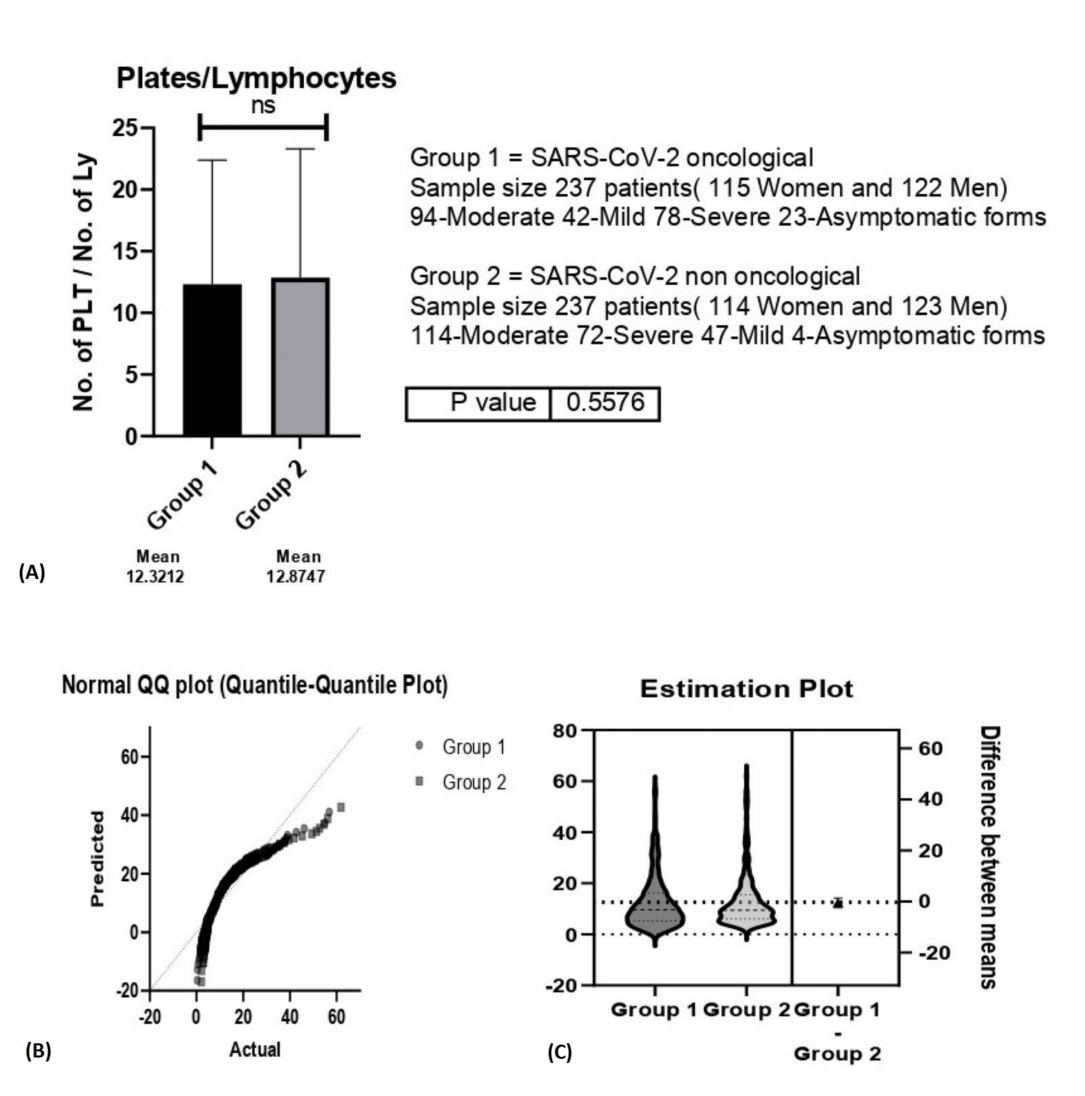
A. Plates/Lymphocytes (P/L) ratio between group 1 (oncological patients with COVID-19) versus group 2 (non-oncological patients with COVID-19); B. Estimation Plot for P/L ratio; C. Normal Quartile-Quartile plot for the P/L ratio. A. ns-p>0.05

Similarly, we observed no statistical significance between the oncological and non-oncological groups when analyzing the P/L ratios (p=0.55) (Figure 6).

**Figure 7:**
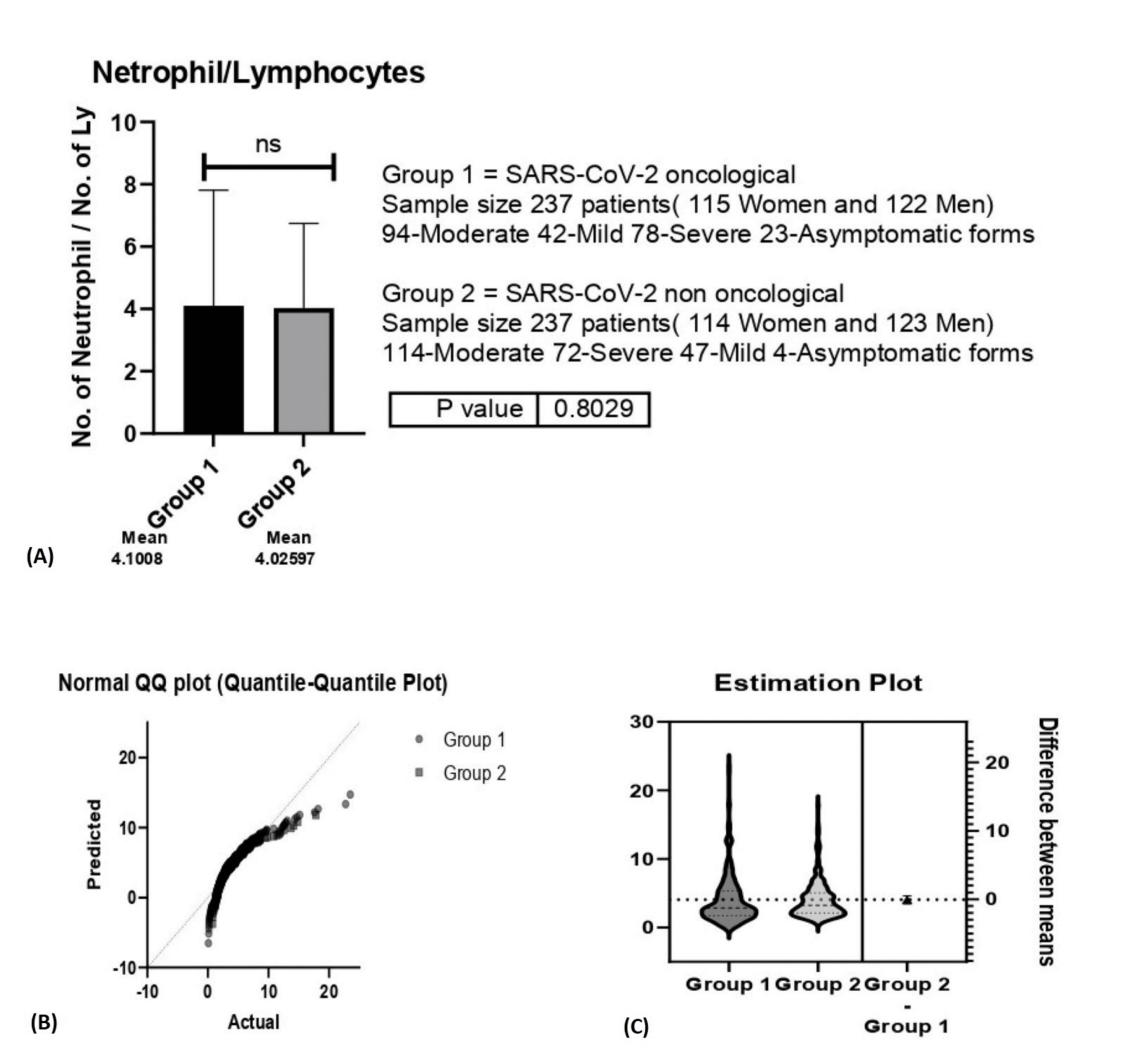
A. Neutrophil/Lymphocyte (N/L) ratio between group 1 (oncological patients with COVID-19) versus group 2 (non-oncological patients with COVID-19); B. Estimation Plot for N/L ratio; C. Normal Quartile-Quartile plot for the P/L ratio. A. ns-p>0.05

The N/L ratio yielded the same results, with no statistically significant differences between the two groups (p=0.8) (Figure 7).

CRP levels were comparable between the group with malignant tumors versus the control group (mean values 4.615 mg/dl versus 4.78 mg/dl). Consequently, no statistically significant differences were observed between the two groups (p>0.05). When comparing PCT levels, we observed a difference between the mean levels for oncological patients (1.6 µg/L) versus non-oncological patients (0.38 µg/L). However, the results were not statistically significant (p>0.05).

Next, we analyzed blood urea and creatinine levels. We observed no statistically significant differences between patient groups when regarding urea levels with mean values being similar (46 mg/dl for oncological patients versus 44 mg/dl for non-oncological patients). As for creatinine levels, we discovered a strong difference between non-oncological patients who displayed a mean level of 2.76 mg/dl versus oncological patients who had markedly lower mean levels of 1.18 mg/dl (p <0.00001).

When comparing blood glucose levels, we observed that non-oncological patients had slightly more elevated mean levels (139 mg/dl) versus oncological patients (127 mg/dl), with the difference being statistically significant (p=0.02). Similarly, non-oncological patients had markedly higher SGPT mean values (150 UI/L) when compared to oncological patients (41 UI/L), the comparison displaying high levels of statistical significance (p<0.0008).

**Figure 8:**
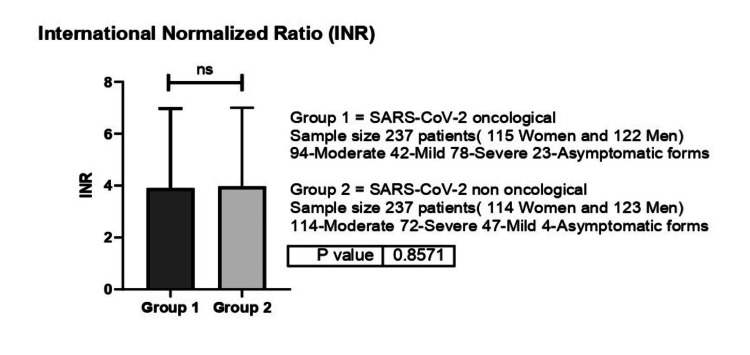
International Normalized Ratio (INR) values between group 1 (oncological patients with COVID-19) versus group 2 (non-oncological patients with COVID-19) ns-p>0.05

Coagulation parameters were similar between the oncological and non-oncological patients. As such, INR, PT and PI values were similar between the two groups, with DD values being the only ones significantly different between groups. As such, INR values were 1.31 for non-oncological patients while oncological patients presented values of 1.38 (p=0.85) (Figure 8).

**Figure 9:**
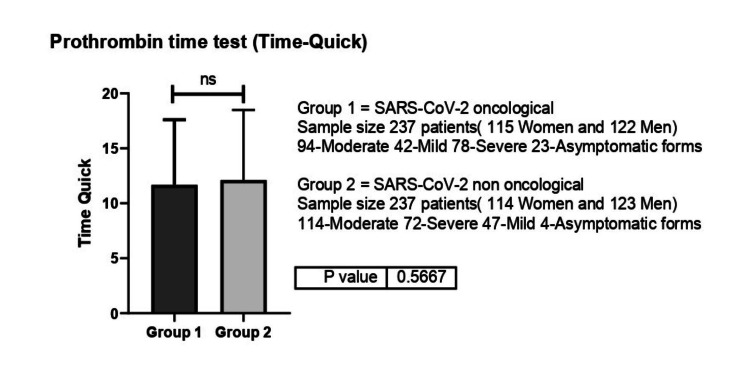
Prothrombin Time (PT) values between group 1 (oncological patients with COVID-19) versus group 2 (non-oncological patients with COVID-19). ns-p>0.05

Similarly, PT values for non-oncological patients were 14.61s versus 15.54s for patients previously diagnosed with cancer (p=0.39) (Figure 9), while PT values were 77.85% for non-oncological patients and 73.7% for oncological patients. DD values were the only coagulation parameters significantly different between oncological (1314 ng/mL) versus non-oncological patients (837.4 ng/mL) (p=0.006).

**Figure 10:**
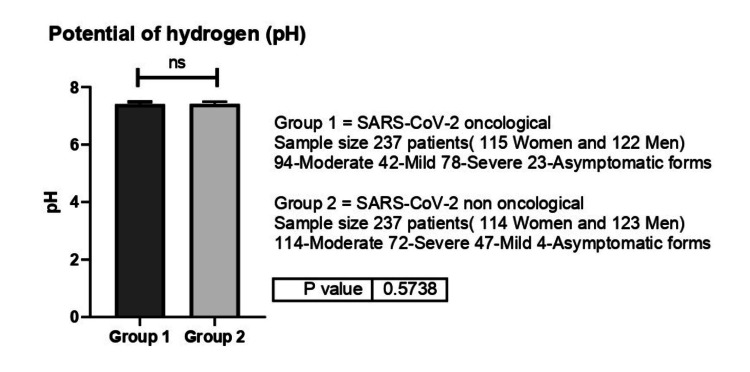
pH values between group 1 (oncological patients with COVID-19) versus group 2 (non-oncological patients with COVID-19). ns-p>0.05

We did not observe any statistically significant differences between the two groups in terms of electrolytes and pH values (Figure 10) (p=0.57).

**Figure 11:**
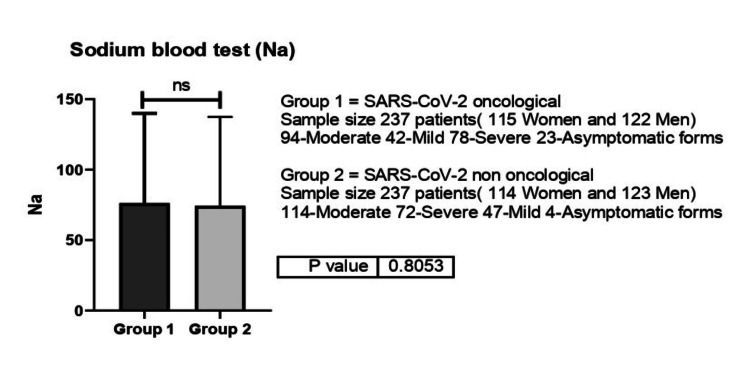
Sodium (Na) values between group 1 (oncological patients with COVID-19) versus group 2 (non-oncological patients with COVID-19). ns-p>0.05

As such, the Na values were slightly more elevated for non-oncological patients (135.6 mEq/l), while oncological patients had slightly lower values (131. 9 mEq/l) (p=0.8) (Figure 11).

**Figure 12:**
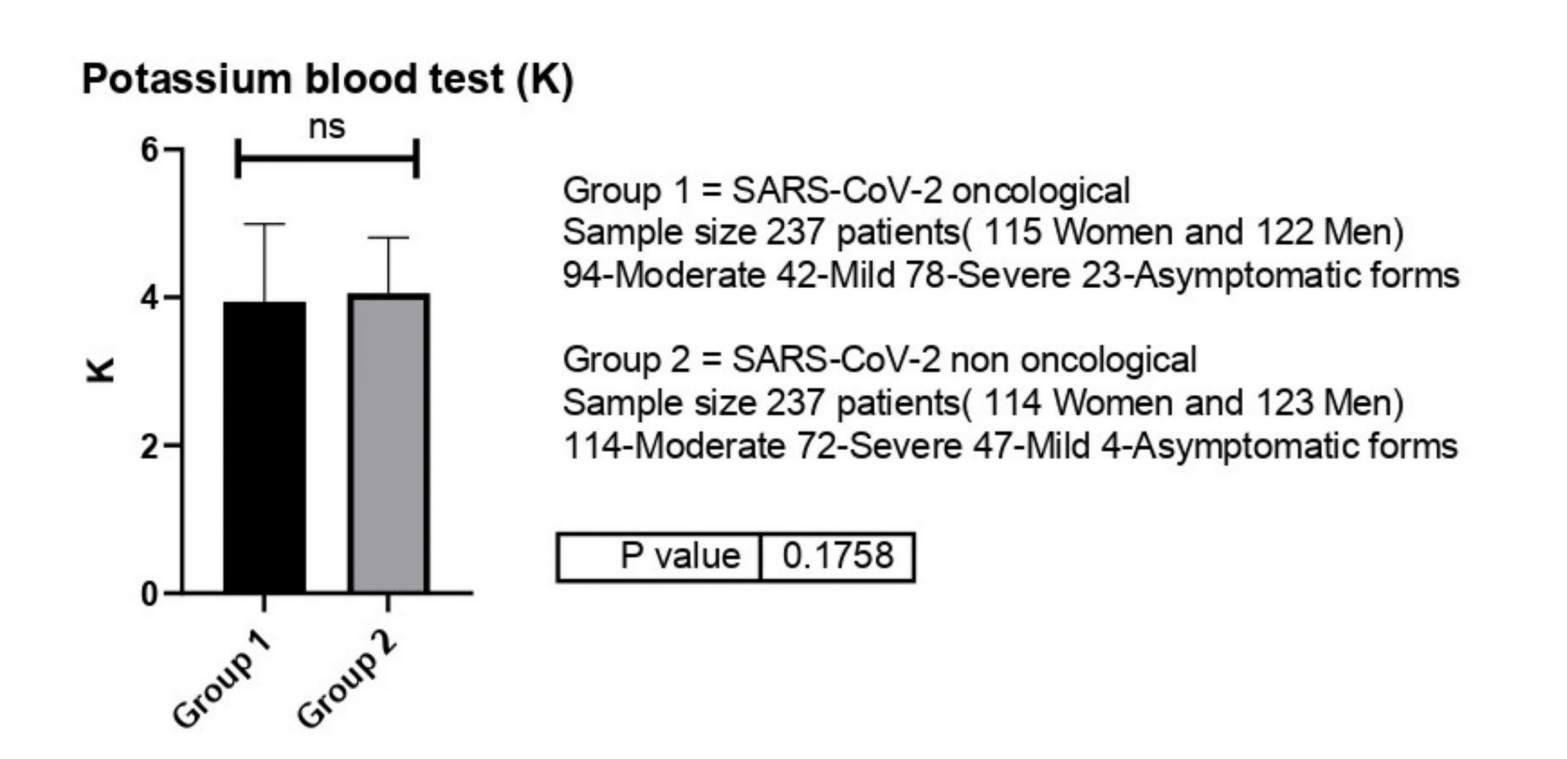
Potassium (K) values between group 1 (oncological patients with COVID-19) versus group 2 (non-oncological patients with COVID-19). ns-p>0.05

K values were identical between the two groups (4 versus 3.99 mEq/l) (p=0.17) (Figure 12).

## Discussion

The global impact of the COVID-19 pandemic has been profound, affecting individuals across diverse demographics and irrespective of their status [22]. Among the groups facing heightened vulnerability are individuals already burdened by a diagnosis of cancer [4,8,23]. The convergence of these two formidable health challenges presents a multifaceted scenario that demands meticulous exploration.

Cancer patients are particularly vulnerable to viral infections, including COVID-19, due to the compromised immune system caused by both the disease and its aggressive treatments, such as chemotherapy and radiotherapy [24,25]. These treatments can lead to neutropenia, lymphopenia, and other forms of immunosuppression, increasing the risk of severe infection. In addition, the cancer itself can affect immune function, making patients more susceptible to infections and complications [26]. Several studies have shown that cancer patients with COVID-19 have worse outcomes compared to non-cancer patients. The relationship between cancer, its treatments, and COVID-19 infection significantly impacts various biological parameters, leading to an increased risk of morbidity and mortality [27]. For example, elevated levels of inflammatory markers, such as CRP and interleukin-6 (IL-6), have been observed in cancer patients with COVID-19, indicating a heightened inflammatory response [27]. This inflammatory state can exacerbate the severity of COVID-19 and contribute to worse clinical outcomes [28].

The effect of COVID-19 on specific biological parameters in cancer patients is multifaceted. Hematological parameters, such as WBC count, neutrophil count, and lymphocyte count, are often significantly altered in these patients. Studies have reported that cancer patients with COVID-19 tend to have lower lymphocyte counts and higher neutrophil counts compared to non-cancer patients with COVID-19, which is indicative of an impaired immune response [29]. Additionally, anemia and thrombocytopenia are common in cancer patients with COVID-19, further complicating their clinical management [30]. The hypercoagulable state observed in COVID-19 patients is also of particular concern for cancer patients, who are already at an increased risk of thrombosis due to their malignancy and its treatment. Elevated D-dimer levels, a marker of coagulation activation, have been associated with worse outcomes in COVID-19 patients, and this is particularly true for those with cancer [31]. Managing anticoagulation in cancer patients with COVID-19 is complex, requiring careful consideration of the risks and benefits [32].

In terms of organ function, COVID-19 can have profound effects on the cardiovascular and respiratory systems, both of which can be critically impacted in cancer patients. Cardiac biomarkers such as troponin and brain natriuretic peptide (BNP) are often elevated in cancer patients with COVID-19, indicating potential cardiac injury or stress [33]. Respiratory complications are also common, with many cancer patients developing severe pneumonia and acute respiratory distress syndrome (ARDS) as a result of COVID-19 infection [30]. Furthermore, liver function tests are often elevated in cancer patients with COVID-19, suggesting hepatic involvement either directly from the virus or secondary to systemic inflammation and hypoxia [34]. Renal function, as measured by serum creatinine and blood urea nitrogen (BUN), can also be impaired in these patients, possibly due to direct viral effects, dehydration, or nephrotoxic medications used during treatment [35]. The interplay between COVID-19 and cancer also extends to metabolic parameters. Hyperglycemia has been identified as a significant predictor of poor outcomes in COVID-19 patients, and this is exacerbated in cancer patients due to the diabetogenic effects of certain cancer therapies [36]. Glycemic control is crucial in managing these patients, as poor glycemic control has been associated with increased mortality and complications [37]. The significance of this study lies in its exploration of the unique challenges faced by cancer patients during the COVID-19 pandemic. By analyzing clinical outcomes, demographic factors, and laboratory parameters, we aim to contribute valuable insights to the existing body of knowledge. The study's focus on a specific cohort admitted to the coronavirus care unit of the “Victor Babeş” Infectious Disease Hospital in Craiova, Romania, allows for a detailed examination of the impact of COVID-19 on individuals with a medically recorded oncological disease. This exploration is essential not only for enhancing our understanding of the intersection between COVID-19 and cancer but also for informing future strategies aimed at mitigating the dual challenges faced by this vulnerable patient population.

Our study delved into both statistically significant and non-significant parameters, offering an encompassing view of the intricate relationship between COVID-19 and cancer within our studied population.

Overall, the analysis indicates that age significantly differs between oncological and non-oncological SARS-CoV-2 patients, suggesting that it could be a factor influencing the distinction between the two groups. The sex distribution between the oncological and non-oncological patients was similar, which implies that sex is not a differentiating factor between these two patient groups in the context of SARS-CoV-2. Similarly, the p-value for the comparison of COVID-19 severity was 0.23, indicating no statistically significant difference, suggesting that the severity of symptoms is comparable between oncological and non-oncological patients.

Elevations in specific biomarkers such as WBC count, neutrophil levels, D-dimer, alanine transaminase (ALT), and creatinine have been associated with poor prognoses in various conditions, including severe COVID-19 and critical illnesses [38]. High WBC and neutrophil counts, coupled with low lymphocyte counts, have been linked to increased mortality in severe COVID-19 cases, reflecting an overactive immune response that can lead to poor outcomes​ [39]. Elevated D-dimer levels indicate a hypercoagulable state, which is associated with worse outcomes in patients with both COVID-19 and cancer, often leading to complications such as thrombosis​ [39]​. Similarly, increased levels of ALT and creatinine suggest liver and kidney dysfunction respectively, which are also predictors of poor prognosis in critically ill patients​ [39]. While the statistical significance between the two groups has been significant (p<0.00001), the mean values of the two groups do not reflect a profound anemia-inducing effect of COVID-19, sufficient enough to draw conclusions regarding a potential risk to vulnerable patients. The markedly elevated total leukocyte counts (p=0.03) among cancer patients, coupled with significantly lower lymphocyte levels (p<0.00001), highlight the intricate interplay between compromised immune systems in cancer patients and their heightened vulnerability to severe COVID-19 outcomes [40,41]. Conversely, we also observed strong statistically significant differences between oncological and non-oncological patients when we compared T/L, M/L and N/L ratios. Surprisingly, lower creatinine levels in cancer patients (p < 0.00001) introduce intriguing questions about the complex dynamics between COVID-19 and pre-existing oncological conditions, particularly their impact on renal function. The strongly statistically significant higher SGPT levels in non-oncological patients (p<0.008) may imply a hepatotoxic effect of certain therapeutic agents or even an autoimmune hepatitis post-vaccination and warrants a more detailed analysis.

Conversely, parameters with statistical significance, though less pronounced, contribute valuable dimensions to our understanding. The higher total leukocyte counts among cancer patients (p=0.03), paired with lower neutrophil (p<0.05) and lymphocyte levels (p<0.05) highlighted earlier, emphasize potential variations in immune response dynamics, contributing to the complicated management of cancer patients who contracted a COVID infection. The high neutrophil levels could also be attributed to granulocyte colony-stimulating factors as well as prior corticosteroid use. The slightly elevated blood glucose levels in non-oncological patients (p=0.02) suggest nuanced metabolic and coagulation variations between the two groups.

The interplay between coagulation parameters and COVID-19 in patients with cancer presents a complex landscape, necessitating a nuanced understanding of the differential coagulopathy observed in COVID-19 versus non-COVID-19 patients. Coagulation abnormalities, notably characterized by alterations in platelet count, DD levels, PT, and INR, underscore the unique coagulopathy associated with COVID-19, known as COVID-19-associated coagulopathy (CAC) [42,43]​​. Despite this, we observed no statistical differences in terms of INR (p=0.85), PT (p=0.56) and PI (p=0.44) values between the two groups. However, we observed a statistically significant difference (p<0.05) between the two groups, with higher values being displayed by oncological patients (p<0.05) but several studies have already indicated that higher levels of D-dimers are indicative of a poorer prognosis for cancer patients [44,45] and given the high percentage of locally advanced or metastatic cancer patients in our group, no clear correlations can be drawn regarding any synergic role between cancer and COVID-19, in this regard. On the other hand, false positives in cancer patients can result from various factors including infections and inflammation, which can elevate markers like WBC, neutrophils, and D-dimer. These elevated values might not always indicate a worsening cancer prognosis but could reflect the body’s response to other concurrent conditions such as infections or inflammation​ [38].

COVID-19 infection has been associated with significant changes in electrolyte balances, specifically affecting sodium and potassium levels, and potentially impacting pH values indirectly through its systemic effects [46]. Research has identified that patients with severe COVID-19 tend to have lower serum concentrations of sodium, potassium, and calcium. This imbalance is important as it is linked to the severity of the disease, suggesting that monitoring and correcting these electrolytes could be crucial for patient management. Despite these findings, we did not identify major electrolyte imbalances between the two groups with Na, K and pH values being virtually identical.

This comprehensive analysis, encompassing parameters with varying degrees of statistical significance, enriches our understanding of the multifaceted interplay between COVID-19 and cancer, providing a foundation for tailored clinical approaches and interventions. Moreover, this tailored approach must take into consideration the rising prospect of the immune-related therapies that are increasingly employed in cancer therapy [47,48].

Despite the valuable insights provided by this study, certain limitations should be acknowledged to ensure a nuanced interpretation of the findings. Firstly, the single-center nature of the study may limit the generalizability of the results to a broader population. Additionally, the exclusion of patients with benign or borderline tumors may impact the comprehensive understanding of the relationship between COVID-19 and various tumor types. Furthermore, the study lacks detailed information on cancer treatments and their timing concerning COVID-19 diagnosis, factors that could significantly influence outcomes. Additionally, the retrospective design introduces inherent biases and may not capture real-time changes in patient management and outcomes. While our study adds significant information to the understanding of COVID-19 in cancer patients, a cautious approach is necessary in generalizing the findings. Further multi-center, prospective studies are warranted to validate and extend these results, addressing the complexities of the intricate interplay between COVID-19 and various cancer types.

Several limitations of the study can be noted. Firstly, due to the enrollment through the emergency ward, no stratification of patients based on the performance index and comorbidities could be done, in comparison to values displayed prior to COVID-19 infection. Secondly, vaccination status was not included in the study mainly because of a drop-off in vaccination rates in the latter half of 2022, due to the lifting of all restrictions as well as conflicting national health policies throughout the period of enrollment which could have compromised the statistical value of the data involved. Thirdly, due to the limited information obtained from patients enrolled through the emergency ward as well as the highly diversified and spurious types of cancer types (each with vastly different prognosis even in advanced stages) and treatments involved, as well as the schedule and timing of each cancer-related therapeutic protocol, we did not include a statistical analysis based on both cancer staging and treatment protocols. Fourthly, the size of the control group could be a source of limitations and increasing it in further studies to a 1:2 or even a 1:3 ratio might reduce selection bias and increase the power. Another limitation is related to the underlying oncological condition, which can impact the majority of biological parameters we have observed as being altered between the two groups. One way to reduce the impact of this bias would be to compare these parameters during and before hospital admission for a COVID-19 infection. However, due to the admission through the emergency ward and a lack of a centralized cancer patient database readily accessible to practitioners, it is very difficult to obtain previous clinical and biological data from patients.

## Conclusions

In conclusion, this study provides a nuanced understanding of the intricate interplay between COVID-19 and cancer, shedding light on the multifaceted challenges faced by this vulnerable patient population. The statistically significant differences observed in various hematological and biochemical parameters between oncological and non-oncological patients underscore the need for tailored clinical approaches. While our findings contribute valuable insights into the unique vulnerabilities of cancer patients during the COVID-19 pandemic, the single-center design and retrospective nature of the study highlight the necessity for further multi-center, prospective research to validate and extend these results.

Moreover, the lack of detailed information on cancer treatments and their timing, calls for comprehensive future studies. Addressing these limitations will be crucial in refining our understanding of the dynamic relationship between COVID-19 and cancer, ultimately informing better clinical management and intervention strategies. While no irrefutable evidence was observed between the two groups, this study lays a foundation for such future endeavors, emphasizing the importance of continuous exploration and adaptation in the face of evolving healthcare challenges.
